# Stroke Recovery in Rats after 24‐Hour–Delayed Intramuscular Neurotrophin‐3 Infusion

**DOI:** 10.1002/ana.25386

**Published:** 2018-12-28

**Authors:** Denise A. Duricki, Svetlana Drndarski, Michel Bernanos, Tobias Wood, Karen Bosch, Qin Chen, H. David Shine, Camilla Simmons, Steven C. R. Williams, Stephen B. McMahon, David J. Begley, Diana Cash, Lawrence D. F. Moon

**Affiliations:** ^1^ Neurorestoration Group Wolfson Centre for Age‐Related Diseases, King's College London London United Kingdom; ^2^ Centre for Integrative Biology King's College London London United Kingdom; ^3^ Blood‐Brain Barrier Group Institute of Pharmaceutical Science, King's College London London United Kingdom; ^4^ Neuroimaging Research Group King's College London London United Kingdom; ^5^ Center for Cell and Gene Therapy, Department of Neuroscience Baylor College of Medicine Houston TX

## Abstract

**Objective:**

Neurotrophin‐3 (NT3) plays a key role in the development and function of locomotor circuits including descending serotonergic and corticospinal tract axons and afferents from muscle and skin. We have previously shown that gene therapy delivery of human NT3 into affected forelimb muscles improves sensorimotor recovery after stroke in adult and elderly rats. Here, to move toward the clinic, we tested the hypothesis that intramuscular infusion of NT3 protein could improve sensorimotor recovery after stroke.

**Methods:**

Rats received unilateral ischemic stroke in sensorimotor cortex. To simulate a clinically feasible time to treatment, 24 hours later rats were randomized to receive NT3 or vehicle by infusion into affected triceps brachii for 4 weeks using implanted catheters and minipumps.

**Results:**

Radiolabeled NT3 crossed from the bloodstream into the brain and spinal cord in rodents with or without strokes. NT3 increased the accuracy of forelimb placement during walking on a horizontal ladder and increased use of the affected arm for lateral support during rearing. NT3 also reversed sensory impairment of the affected wrist. Functional magnetic resonance imaging during stimulation of the affected wrist showed spontaneous recovery of peri‐infarct blood oxygenation level–dependent signal that NT3 did not further enhance. Rather, NT3 induced neuroplasticity of the spared corticospinal and serotonergic pathways.

**Interpretation:**

Our results show that delayed, peripheral infusion of NT3 can improve sensorimotor function after ischemic stroke. Phase I and II clinical trials of NT3 (for constipation and neuropathy) have shown that peripheral high doses are safe and well tolerated, which paves the way for NT3 as a therapy for stroke. **ANN NEUROL 2019;85:32–46.**

Ischemic stroke occurs in the brain when blood flow is restricted, causing brain cells to die rapidly. Worldwide, there are an estimated 31 million stroke survivors, with another 9 million new strokes annually. Therapies that reverse impairments are urgently needed.^1^

Neurotrophin‐3 (NT3) is a growth factor that plays a key role in the development and function of locomotor circuits, including descending serotonergic^2^ and corticospinal tract (CST) axons^3^ and afferents from muscle and skin that mediate proprioception and tactile sensation.^4^, ^5^, ^6^ However, levels of NT3 drop in the postnatal period.^7^ We and others have shown that delivery of NT3 into the central nervous system (CNS) promotes recovery in models of spinal cord injury,^8^, ^9^, ^10^, ^11^, ^12^, ^13^, ^14^, ^15^ but this involved invasive routes of delivery or gene therapy. We also recently showed that injection of an adeno‐associated viral vector (AAV) encoding full‐length human NT3 (preproNT3, 30 kDa) into forelimb muscles 24 hours after stroke in adult or elderly rats in adult rats improved CST sprouting and sensorimotor recovery.^16^ We had originally expected that AAV1 would be trafficked from muscle to the spinal cord retrogradely in axons^17^ and that enhanced secretion of NT3 by motor neurons would lead to sprouting of the spared CST^9^, ^18^, ^19^ and sensorimotor recovery. However, although NT3 protein was overexpressed in injected muscles, to our surprise we found little evidence for expression of the human NT3 transgene in the spinal cord or cervical dorsal root ganglia (DRGs)^14^, ^16^ using this modest dose of AAV. This serendipitous result led us to reject our original assumption that sensorimotor recovery required expression of the human *NT3* transgene in the spinal cord and to wonder whether intramuscular infusion of NT3 protein would suffice.

Accordingly, here, we tested the hypothesis that infusion of NT3 protein (14 kDa) into impaired forelimb muscles would improve sensorimotor recovery after stroke (ie, bypassing the need for gene therapy and spinal surgery). This is plausible because others have shown that a signal from muscle spindles can improve neuroplasticity of descending pathways and can enhance recovery after spinal cord lateral hemisection.^20^ Notably, NT3 protein is synthesized by muscle spindles^21^ and NT3 can be transported from muscle to sensory ganglia and spinal motor neurons in nerves^6^, ^14^, ^16^ as well as to the CNS and peripheral nervous system (PNS) via the bloodstream.^22^, ^23^, ^24^ Importantly, NT3 protein has excellent translational potential; phase I and II clinical trials have shown that repeated, systemic high doses of NT3 protein are well tolerated, safe, and effective in >200 humans with sensory and motor neuropathy (Charcot–Marie–Tooth type 1A)^25^ or constipation including in people with spinal cord injury.^25^, ^26^ These studies pave the way for NT3 as a therapy for stroke in humans. We now show in a blinded, randomized preclinical trial that treatment of impaired upper arm muscles with human NT3 protein reverses sensory and motor disability in rats when treatment is initiated in a clinically feasible timeframe (24 hours after stroke).

## Materials and Methods

Full methodological details are available online.^27^

### *Experimental Design*

All surgical procedures and behavioral testing were performed using a randomized block design with experimenters and principal investigator blinded to treatment groups by a third party.^27^ All procedures were in accordance with the Animals (Scientific Procedures) Act of 1986. All protocols involving animals received prior approval by the King's College London Animal Welfare Ethical Review Board and were authorized by the UK Home Office Project (license number 70/7865, held by L.D.F.M.).

### *Stroke or Sham Lesions*

**Figure 1:**
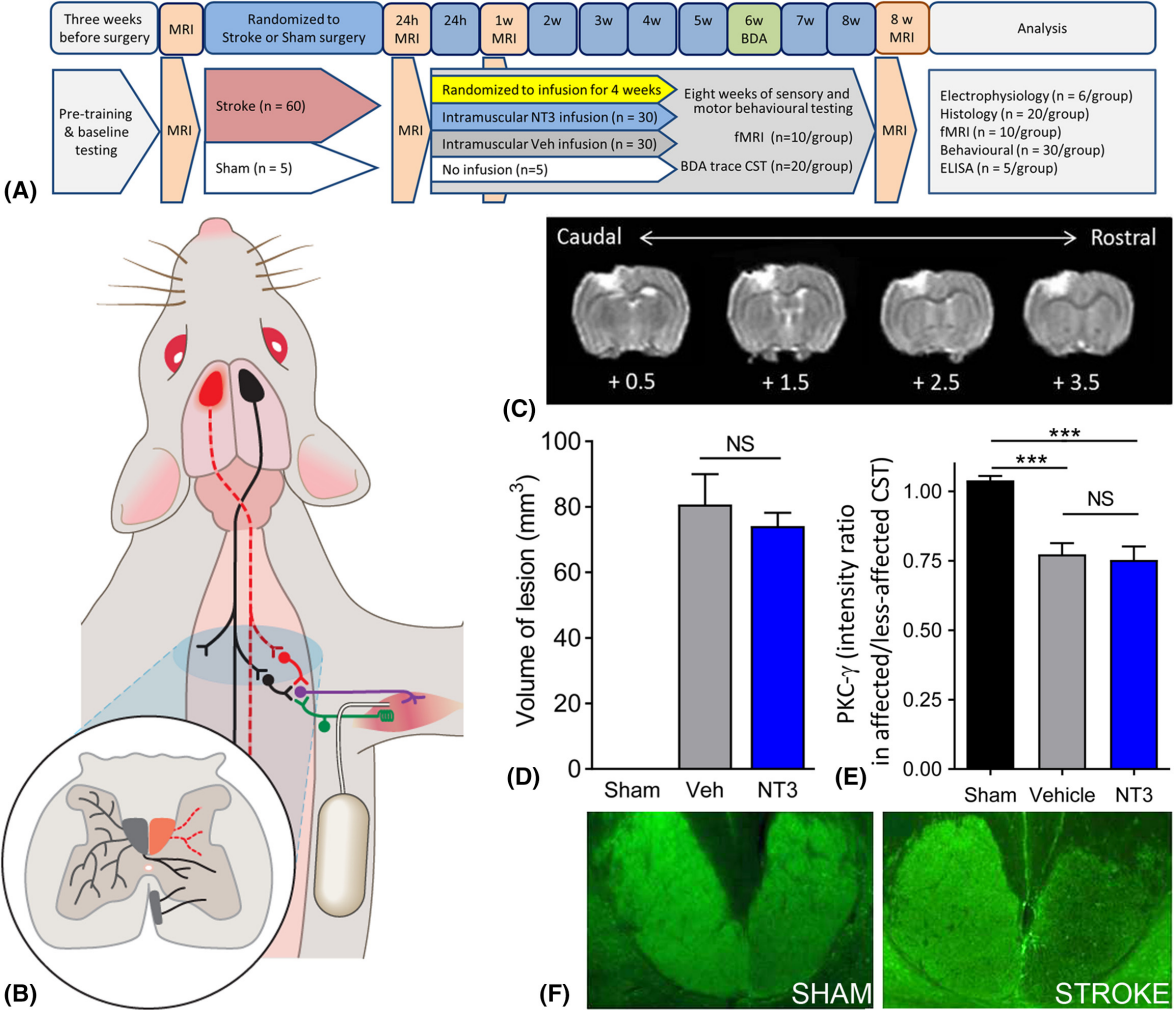
Focal stroke caused unilateral infarction in sensorimotor cortex, resulting in a loss of corticospinal axons. (A, B) Rats received sham surgery (n = 5) or unilateral cortical stroke, and 24 hours later infusion of neurotrophin‐3 (NT3; n = 30) or vehicle (Veh; n = 30) into the affected triceps brachii was initiated for 1 month. Six weeks after stroke, anterograde tracer was injected into the contralesional sensorimotor cortex. Rats underwent 8 weeks of behavioral testing. Structural magnetic resonance imaging (MRI) was conducted on all rats at 24 hours, 1 week, and 8 weeks after stroke and functional MRI (fMRI) was conducted in a subset of rats at baseline, week 1, and week 8. Electrophysiology was performed in the subset of rats that did not receive biotinylated dextran amine (BDA) tracing. All surgeries, treatments, and behavioral testing were performed using a randomized block design, and the study was completed blinded to treatment allocation. (C) T2 MRI scans 24 hours after stroke, immediately prior to treatment, showing infarct in coronal sections rostral (numbers represent millimeters) to bregma. (D) There were no differences between stroke groups in mean lesion volume at 24 hours (Mann–Whitney *p* value = 0.86). (E) Photomicrographs showing loss of corticospinal tract (CST) axons in the upper cervical dorsal columns 8 weeks after stroke (right) relative to sham surgery (left), visualized using protein kinase C gamma (PKC‐γ) immunofluorescence. ***p ≤ 0.001. (F) Stroke caused a significant loss of CST axons relative to shams (Kruskal–Wallis χ^2^ = 12.0, *p* = 0.002; Mann–Whitney *p* values <0.001), with no differences between vehicle‐treated and NT3‐treated rats (Mann–Whitney *p* = 0.96). Representative images of 1 stroke and 1 sham animal are shown. Panel B is modified from Duricki et al^16^ with permission. ELISA = enzyme‐linked immunosorbent assay; NS = not significant. [Color figure can be viewed at www.annalsofneurology.org]

Sixty Lister Hooded outbred female rats (~4 months old, 200–300 g, Charles River, Wilmington, MA) received focal ischemic stroke in the sensorimotor cortex representing the dominant forelimb (Fig 1), defined by the cylinder test, as described for elderly rats^16^ (see Fig 1A, B) with minor modifications as described in detail elsewhere.^27^ Briefly, anesthetized animals were transferred to a stereotaxic frame where a midline incision was made, the cortex was then exposed via craniotomy using the following coordinates (defined as anteroposterior [AP] and mediolateral [ML]): AP 4 mm to −2 mm, ML 2 mm to 4 mm, relative to bregma. Endothelin‐1 (ET‐1; 400 pmol/μl in sterile saline; Calbiochem, San Diego, CA) was applied using a glass micropipette attached to a Hamilton syringe. One microliter of ET‐1 was applied to the overlying dura to reduce bleeding, and immediately thereafter, the dura mater was incised and reflected. Four 1 μl volumes of ET‐1 were administered topically, and four 1 μl volumes were microinjected intracortically (at a depth of 1 mm from the brain surface) at the following coordinates (from bregma and midline, respectively): (1) AP 3.5 mm, ML 2.8 mm; (2) AP 2.0 mm, ML 2.8 mm; (3) AP 0.5 mm, ML 2.8 mm; and (4) AP −1.0 mm, ML 2.8 mm. Temperature was maintained using a rectal probe connected to a homeothermic blanket (Harvard Apparatus, Holliston, MA) placed under the animal, which maintained rectal temperature at 36 ± 1°C. Prior to suturing, the animal was left undisturbed for 5 minutes. The skull fragment was then replaced and sealed using bone wax (Covidien, Gosport, UK). Five sham‐operated rats received all procedures excluding craniotomy. All rats survived this surgery. Animals were given buprenorphine (0.01 mg/kg, subcutaneously).

### *Structural and Functional Magnetic Resonance Imaging*

Structural magnetic resonance imaging (MRI) images were obtained prior to stroke, 24 hours after stroke, and at 1 and 8 weeks after stroke as described with full details elsewhere.^16^, ^27^ Functional MRI (fMRI) was performed prior to stroke and at 1 and 8 weeks after stroke during non‐noxious stimulation of the affected wrist as described fully elsewhere^27^ in a subset of rats that did not receive intracortical injections of biotinylated dextran amine (BDA) tracer (n = 10/group). Two animals died following alpha chloralose anesthesia due to breathing difficulties in recovery.

### *NT3 or Vehicle Treatment*

Twenty‐four hours after stroke (immediately after MRI), rats were randomized to treatment using NT3 or vehicle, infused into affected triceps brachii muscles for 1 month via thin, flexible implanted catheters and subcutaneous osmotic minipumps (2ML2; Alzet, Cupertino, CA; 5 μl/h flow rate, 2 ml reservoir) compatible with MRI.^27^ Full technical details have been provided elsewhere.^27^ Treatments were vehicle (0.9% saline containing 0.1% bovine serum albumin; Sigma, St Louis, MO; A3059; 9048‐46‐8) or vehicle containing recombinant human NT3 (100 μg/ml; gift of Genentech, South San Francisco, CA). Pumps were replaced after 2 weeks and removed after 4 weeks. Skin was sutured and analgesic was administered as above. All rats survived this surgery. Pilot experiments showed that the pump flow rate (5 μl/h) was sufficient to deliver substances (0.9% saline containing 1% Fast Green, Sigma) to the entire volume of the triceps muscles. The identity of this preparation of NT3 was confirmed to consist of the ~14 kDa monomer and ~28 kDa dimer by sodium dodecyl sulfate–polyacrylamide gel electrophoresis and liquid chromatography–mass spectrometry.^27^ The NT3 dose (12 μg per 24 hours) was selected based on previous experiments.^28^

### *Enzyme‐Linked Immunosorbent Assay*

A random subset of rats (n = 5/group) were anesthetized (4 weeks following stroke, before pumps were removed) and triceps brachii and C7 spinal cord were rapidly dissected and snap frozen in liquid nitrogen prior to storage at −80°C. Tissue was homogenized in ice‐cold lysis buffer containing 137 mM NaCl, 20 mM Tris‐HCl (pH 8.0), 1% NP40, 10% glycerol, 1 mM phenylmethanesulfonylfluoride, 10 μg/ml aprotinin, 1 μg/ml leupeptin, and 0.5 mM sodium vanadate, using 10 μl buffer per milligram of tissue. Protein content was measured and NT3 enzyme‐linked immunosorbent assay (ELISA) was carried out according to manufacturers’ instructions (Emax; Promega, Madison, WI). Full details have been given elsewhere.^27^

### *Behavioral Testing*

Rats were handled and trained for 3 weeks on the horizontal ladder before the study began. Preoperative baseline scores for the horizontal ladder, the vertical cylinder, and the grip strength test were collected 1 week before surgery.

#### Assessment of Somatosensory Responsiveness

**Figure 2:**
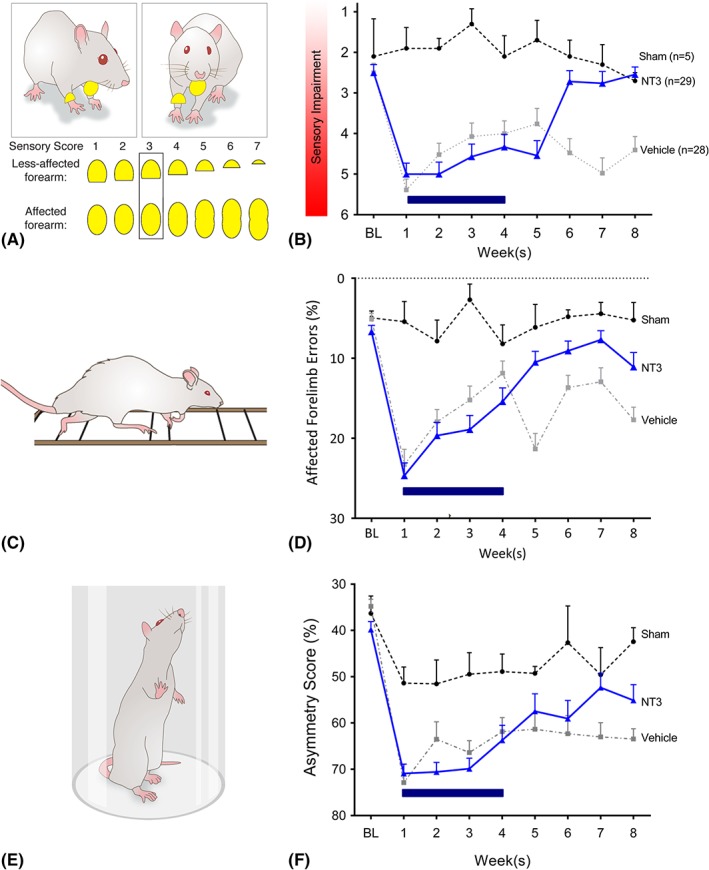
Delayed neurotrophin‐3 (NT3) treatment improved responsiveness to somatosensory stimulation, improved walking, and partially restored use of the affected forelimb for lateral support during rearing. (A) Somatosensory deficits were assessed using pairs of adhesive patches attached to the rat's wrists. (B) Treatment with NT3 caused improvement compared to vehicle (linear model; *F*_2, 126_ = 32.5, *p* < 0.001; post hoc *p* = 0.003), whereas vehicle‐treated stroke rats showed a deficit relative to sham rats that persisted for 8 weeks (linear model; *F*_14, 263_ = 4.1, *p* < 0.001; post hoc *p* values <0.02). NT3 caused improvements such that by 6 weeks they had recovered fully relative to shams (post hoc *p* values >0.4) and to vehicle‐treated stroke rats (post hoc *p* values <0.001). There were no differences between NT3‐treated and vehicle‐treated rats at 1 week (*t* test *p* = 0.33). BL = baseline. (C) Accuracy of paw placement by the affected forelimb during walking was assessed using a horizontal ladder with irregularly spaced runs. (D) One week after stroke, NT3‐treated and vehicle‐treated rats made a similar number of misplaced steps with their affected forelimb (*t* test *p* = 0.78), expressed as a percentage of total steps by that limb. Importantly, the NT3 group progressively recovered compared to the vehicle group (group *F*_2, 52_ = 17.0, *p* < 0.001; post hoc *p* = 0.008) and differed from the vehicle group from weeks 5 to 8 (group × time *F*_14, 355_ = 6.2, *p* < 0.001; post hoc *p* values <0.005); whereas the vehicle group remained impaired relative to shams from weeks 5 to 8 (*p* values <0.006), from weeks 5 to 8 the NT3 group made nearly as few errors as shams (post hoc *p* values >0.05). (E) The vertical cylinder test assessed use of the affected forelimb for lateral support during rearing. (F) Stroke caused a reduction in the use of the affected forelimb during rearing in a vertical cylinder in both NT3‐treated and vehicle‐treated rats relative to shams (group *F*_2, 56_ = 6.1, *p* = 0.004; post hoc *p* values = 0.001 and 0.003, respectively) with no differences between stroke groups at 1 week (*p* = 0.52). NT3 treatment caused a progressive recovery in the use of the affected forelimb (group × wave *F*_14, 356_ = 1.78, *p* = 0.04) by weeks 7 and 8 relative to vehicle‐treated rats (*p* values = 0.015 and 0.067, respectively). Sham group, n = 5; stroke and NT3 group, n = 29; stroke and vehicle group, n = 28. Sham rats are represented by circles with dashed lines, NT3 stroke rats are represented by triangles with solid lines, and vehicle stroke rats are represented by squares with dotted lines. [Color figure can be viewed at www.annalsofneurology.org]

The “adhesive patch” test was used to measure (1) the time taken to contact stimuli on the wrists, (2) the time taken to remove stimuli from the wrists, and (3) the magnitude of lack of responsiveness to stimuli on the affected wrist as previously described.^16^, ^27^ To determine whether the rats preferentially removed a sticker from their less‐affected wrist before their more‐affected wrist, the order and side of label removal was recorded. This was repeated 4 times per session until a > 75% preference had been found; if this was not the case, a fifth trial was conducted. The magnitude of asymmetry was established using the 7 levels of stimulus pairs on both wrists as previously described (Fig 2A). From trial to trial, the size of the stimulus was progressively increased on the affected wrist and decreased on the less affected wrist by an equal amount (14.1mm^2^), until the rat removed the stimulus on the affected wrist first (reversal of original bias). The higher the score, the greater the degree of somatosensory impairment.

#### Walking

To assess impairments in forelimb and hindlimb function after stroke, rats were videotaped as they walked along a horizontal ladder.^16^, ^27^ Rats were videotaped crossing a horizontal ladder (1 m) with irregularly spaced rungs weekly, 3 times per session. Any slight paw slips, deep paw slips, and complete misses were scored as an error. The mean number of errors per step was calculated for each limb for each week.

#### Assessment of Forelimb Use During Rearing

The cylinder test was used to assess asymmetries in forelimb use for postural support during rearing within a transparent 20 cm‐diameter and 30 cm‐high cylinder.^16^, ^27^ An angled mirror was placed behind the cylinder to allow movements to be recorded when the animal turned away from the camera. During exploration, rats rear against the vertical surface of the cylinder. The first forelimb to touch the wall was scored as an independent placement for that forelimb. Subsequent placement of the other forelimb against the wall to maintain balance was scored as “both.” If both forelimbs were simultaneously placed against the wall during rearing, this was scored as “both.” A lateral movement along the wall using both forelimbs alternately was also scored as “both.” Scores were obtained from a total number of 10 full rears to control for differences in rearing between animals. Once scores had been acquired, forelimb asymmetry was calculated using the formula: 100 × (ipsilateral forelimb use +1/2 bilateral forelimb use) / total forelimb use observations.

### *Anatomical Tracing*

To visualize uninjured CST axons, 6 weeks after stroke, 10% BDA in phosphate‐buffered saline (PBS; pH 7.4) was microinjected unilaterally into the uninjured sensorimotor cortex as described previously.^16^, ^27^ Briefly, animals were placed in a stereotaxic frame and 6 burr holes were made into the skull at the following coordinates: (1) AP 1 mm, ML 1.5 mm; (2) AP 0.5 mm, ML 2.5 mm; (3) AP 1.5 mm, ML 2.5 mm; (4) AP 0.5 mm, ML 3.5 mm; (5) AP 2.0 mm, ML 3.5 mm; and (6) AP − 0.5 mm, ML 3.5 mm, relative to bregma. At each site, 0.5 μl injections of BDA (10% in PBS) were delivered using a glass micropipette attached to a Hamilton syringe inserted 2 mm from the skull surface and delivered at a rate of 0.25 μl/min. Animals were left for 2 weeks before being perfused. Tract tracing was not performed in rats that were to undergo fMRI or neurophysiology.

### *Neurophysiology*

We recorded from the ulnar nerve on the affected side and stimulated, in the pyramids, the CST corresponding to the affected or less‐affected hemisphere, as described previously.^27^ At the end of the study, 18 rats (6 rats per group) were anesthetized with an intraperitoneal injection of 1.25 g/kg urethane (Sigma‐Aldrich, St Louis, MO). The rats were kept at 37°C with a homeothermic blanket system and rectal thermometer probe. Tracheotomy was performed, and a tracheal cannula was inserted. The pyramids were then exposed ventrally by blunt dissection and removal of a small area of bone. The brachial plexus of the affected forelimb was exposed from a ventral approach by dissecting the pectoralis major. The ulnar and median nerves were dissected free from surrounding connective tissue and cut distally (to prevent twitches of target muscles). Skin flaps from the incision formed a pool, which was filled with paraffin oil. The proximal segment of the ulnar nerve was mounted on a pair of silver wire hook electrodes (with >1 cm separation). The concentric bipolar stimulation electrode (CBBPC75; FHC, Bowdoin, ME) was located 1 mm lateral to the midline and gently lowered through the pyramid up to a maximum depth of 1.5 mm while stimulating at 300 μA (4 pulses, square wave pulse width = 100 microseconds, frequency = 500 Hz). At the electrode location providing maximal ulnar nerve response, stimuli of increasing intensity were applied in the range of 50 to 400 μA, in 50 μA increments. Five sweeps were captured at each stimulus intensity. The signal was rectified, and the area under the curve was measured between 17 and 45 milliseconds. Data were analyzed using 2‐way repeated measures analysis of variance (ANOVA). Graphs show mean and standard error of the mean for the area under the curve for stimuli given at 400 μA.

### *Histology*

Eight weeks after stroke surgery and 2 weeks after injection of BDA, rats were terminally anesthetized with sodium pentobarbital (80 mg/kg intraperitoneal) and perfused with PBS for 5 minutes, followed by 500 ml of 4% paraformaldehyde in PBS for 15 minutes. The brain, C6–C8 spinal cord, C7 and C8 DRGs, and both arms were carefully dissected and stored in 4% paraformaldehyde in PBS for 2 hours and then transferred to 30% sucrose in PBS and stored at 2 to 5°C. Spinal cord segments C1 and C7 was embedded in OCT and 40 μm transverse slices were cut using a freezing stage microtome. Ten series of sections were collected.

Series of 40 μm‐thick transverse sections of fixed spinal cord were immunolabeled as previously described.^16^, ^27^ Primary antibodies (overnight) were rabbit anti–protein kinase C gamma (PKCγ; 1:500, sc‐211; Santa Cruz Biotechnology, Santa Cruz, CA) and rabbit antiserotonin (1:6,000, #20080; ImmunoStar, Hudson, WI). Secondary antibodies (3 hours) were: goat antirabbit IgG Alexa 488 (1:1,000; Invitrogen, Carlsbad, CA) with DAPI (1:50,000, Sigma).

**Figure 3:**
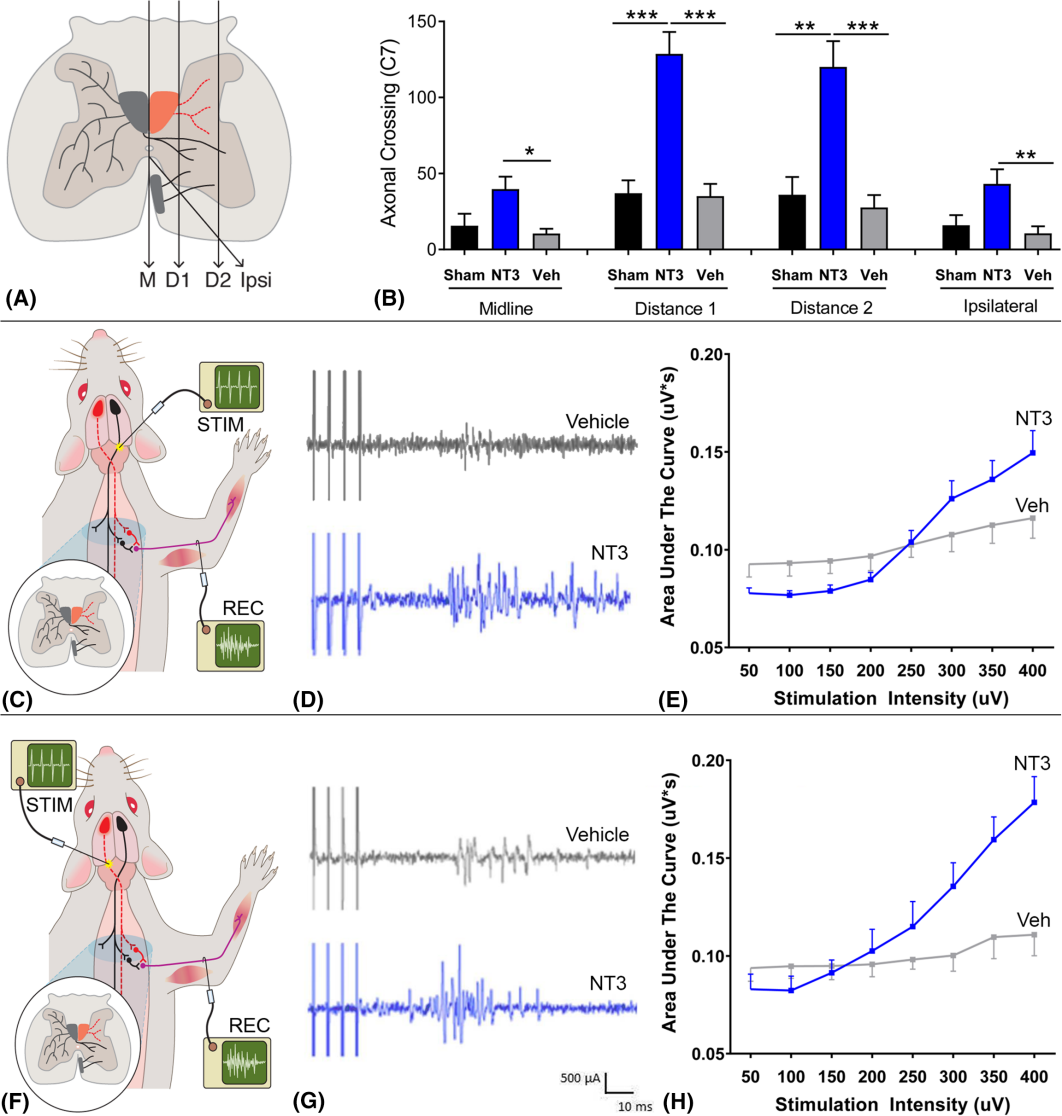
Delayed neurotrophin‐3 (NT3) treatment caused corticospinal tract (CST) sprouting and increased output in forelimb motor nerves during CST stimulation. (A) Corticospinal axons were anterogradely traced from the less‐affected cortex and were counted at the midline (M), at 2 more lateral planes (D1 and D2), and crossing into gray matter from the ipsilateral, ventral tract (Ipsi). (B) NT3 treatment caused an increase in the number of axons crossing at the midline (*F*_2, 28_ = 6.1, *p* = 0.007; post hoc *p* value = 0.003), at 2 lateral planes denoted as D1 (*F*_2, 28_ = 20.3, *p* < 0.001, post hoc *p* value<0.001) and D2 (*F*_2, 28_ = 13.8,*p* < 0.001, post hoc *p* value<0.001), and from the ventral CST on the treated side (*F*_2, 28_ = 5.2, *p* = 0.013; post hoc *p* value = 0.005). Ten rats per group were used for tract tracing. (C–E) The CST from the less‐affected hemisphere or (F–H) lesioned hemisphere was stimulated in the medullary pyramids (before the decussation) and the motor output was recorded from the ulnar nerve on the treated side. (D, G) The majority of spikes were detected between 17 and 45 milliseconds, latencies consistent with polysynaptic transmission, in both vehicle‐treated rats (top trace in D and G) and NT3‐treated rats (bottom trace in D and G) when the less‐affected or affected CST was stimulated. (E, H) Stimulus intensity was increased incrementally from 50 to 400 μA and the area under the curve were measured (between 17 and 45 milliseconds) after stimulation of the affected or less‐affected CST. NT3 treatment caused increased output in the ulnar nerve during stimulation of either the affected CST (2‐way repeated measures analysis of variance intensity × group interaction *F*_1, 14_ = 7.9, *p* = 0.008, n = 6 vehicle, n = 6 NT3) or less‐affected CST (*F*_2, 17_ = 9.8, *p* = 0.01, n = 4 vehicle, n = 6 NT3). *p ≤ 0.05, **p ≤ 0.01, ***p ≤ 0.001. REC = recording; STIM = stimulating; Veh = vehicle. Panels A, C, and F were modified from reference 16 with permission. [Color figure can be viewed at www.annalsofneurology.org]

BDA staining was performed as described before.^16^, ^27^ CST axons were counted that crossed the midline, at 2 more lateral planes and at an oblique plane (Fig 3A) on the affected side at C7 and C1. For each rat, we estimated the number of CST axons per cord segment by calculating the average number of CST axons per section and then multiplying by a scaling factor (number of sections per segment).

**Figure 4:**
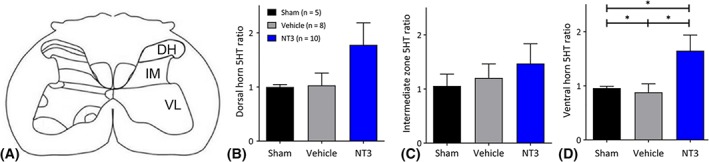
Neurotrophin‐3 (NT3) treatment caused plasticity of serotonergic axons in the C7 spinal cord. (A) The total length of serotonergic arbors was measured in 3 regions (DH = dorsal horn; IM = intermediate horn; V = ventral horn) on the treated side relative to the opposite hemicord. (B) NT3 treatment did not increase serotonergic (5HT) arbor length in the dorsal horn or (C) the intermediate zone but (D) did increase serotonergic arbor length in the ventral horn relative to vehicle (1‐way analysis of variance *F*_2, 22_ = 3.5, *p* = 0.05; Bonferroni post hoc test *p* = 0.025). *p ≤ 0.05. [Color figure can be viewed at www.annalsofneurology.org]

The total length of serotonergic processes was measured using a method^14^ that is well suited for quantification of dense terminal arbors. Processes were identified using the “adjust threshold” function in ImageJ, and fiber lengths were measured in 3 areas: the dorsal horn, intermediate horn, and ventral horn (Fig 4B) in 3 sections per rat. We calculated the ratio of the sides ipsilateral and contralateral to treatment for the 3 areas separately.

Immunofluorescence was visualized under a Zeiss (Oberkochen, Germany) Imager.Z1 microscope or a confocal Zeiss LSM 700 laser scanning microscope. Photographs were taken using the AxioCam and AxioVision LE Rel. 4.2 or LSM Image Browser software for image analysis.

### *Radiolabeling of NT3*

NT3 protein was radiolabeled with 50 μCi (1.85 MBq) N‐succinimidyl [2,3‐^3^H]propionate (^3^H‐NSP) and separated from unbound ^3^H‐NSP using an ÄKTAprime (GE Healthcare, Chicago, IL) purification system using a modification of a previous method.^29^ Fifty micrograms of [^3^H]NT3 or [^3^H]albumin was injected intravenously as a 300 μl bolus into 95 adult C57Bl/6 mice (7–8 weeks old). This method had been optimized for mice. After 3, 15, 20, 30, 40, 60, or 90 minutes, brain, spinal cord, and serum were taken for scintillation counting. The rate of influx was calculated from the Patlak plot^30^ of the distribution volume (V_D_) for [^3^H]NT3 and [^3^H]albumin against the plasma area under the curve.

The same treatment was repeated for mice 24 hours after stroke, with an incubation period of 40 minutes (n = 6). ^14^C sucrose (0.074 MBq; vascular marker) was injected toward the end of the incubation, and the brain tissue was taken for capillary depletion analysis. Brain tissue was homogenized in physiological buffer (30 μl/mg of tissue) and 26% dextran (40 μl/mg of tissue) as described previously.^31^ The homogenate was subjected to density gradient centrifugation (5,400 × *g* for 15 minutes at 4°C) to give an endothelial cell‐enriched pellet and a fraction containing the brain parenchyma and interstitial fluid. The samples were solubilized and counted as described above. V_D_ was calculated. The values were corrected for ^14^C sucrose. Data were analyzed for capillary fraction, parenchymal fraction, and whole brain using 1‐way ANOVA and Bonferroni *t* tests.

### *Statistics*

A set of a priori sample size calculations were performed and reported previously for this ET‐1 model and for 4 behavioral tests (Montoya staircase test, adhesive patch test, cylinder test, horizontal ladder; see Methods and Table 1 in Soleman et al^32^). G*Power 3.1 was used to estimate the effect size of the difference between the stroke and sham groups for each behavioral test using the post hoc algorithm for *F* tests (repeated measures, between factors). Next, G*Power was used^33^ to estimate the minimum sample sizes that would be required in experiments (using 2 groups) to identify treatment effects of 3 different magnitudes (25% recovery, 50% recovery, 75% recovery) using the a priori algorithm for *F* tests (repeated measures, between factors) using the following parameters: type I error threshold (α) ≤ 0.05 and power (1 − β) ≥ 0.80. The “correlation between repeated measures” parameter was derived by calculating the mean of the Pearson product‐moment correlation coefficients for each of all possible (6) pairs of (4) time points in that study.^32^ In the present study, we chose n = 30 for the NT3 and vehicle groups to increase our chances of detecting small improvements in sensorimotor function based on these a priori power calculations. We knew from previous work using NT3 what typical effect sizes could be expected in this model.^16^

Statistical analyses were conducted using SPSS (v18.0; IBM, Armonk, NY). Graphs show means ± standard error of the mean, and n denotes number of rats or mice. The threshold for significance was 0.05. Corticospinal tract sprouting data were analyzed using 1‐way ANOVA per plane and unpaired *t* tests. Serotonergic fiber lengths were analyzed by region using 1‐way ANOVA and Bonferroni post hoc tests. Other histology and molecular biology data were assessed using Kruskal–Wallis and Mann–Whitney tests. Behavioral data were analyzed using linear models and restricted maximum likelihood estimation to accommodate data from rats with occasional missing values (using SPSS MIXED with Fisher least squares difference post hoc tests as described previously^34^). Baseline scores were used as covariates. Post hoc *p* values were only reported when the preceding global (protected) test (eg, factorial linear models, 1‐way ANOVAs, or Kruskal–Wallis tests) was significant for the main effect or interaction term (*p* < 0.05). For transparency, post hoc *p* values are stated exactly (or when small, as *p* < 0.001, for example). Degrees of freedom are reported to the nearest integer. Normality was assessed using histograms. Tests were 2‐tailed.

## Results

### *MRI Confirmed Equivalent Infarct Volumes between Stroke Groups*

MRI confirmed that infarcts included the forelimb and hindlimb areas in sensorimotor cortex (see Fig 1). There was no difference in the mean infarct volume between stroke groups at 24 hours or 1 or 8 weeks after stroke. Loss of CST axons was assessed at 8 weeks in the upper cervical spinal cord using PKCγ immunofluorescence.^35^, ^36^ Stroke caused a 24% loss of CST axons in the dorsal columns of the upper cervical spinal cord relative to shams, with no difference between vehicle‐treated and NT3‐treated rats. Together, the MRI and PKCγ histology data indicate that there were no confounding pretreatment differences in mean infarct volumes and that NT3 did not act as a neuroprotective agent, as expected, based on our previous results^16^ and given that treatment was initiated after the majority of cell death would have occurred.

### *Delayed NT3 Improved Forelimb Sensory and Motor Function after Stroke*

An adhesive patch was attached to each wrist of the rat, and the order of their removal was noted. From trial to trial, the size of the patch was progressively increased on the affected wrist and decreased on the less affected wrist by an equal amount (see Fig 2A), until the rat removed the patch on the affected wrist first. A high score (eg, 6) denotes a deficit (ie, that a rat preferentially removed the smaller stimulus from their less‐affected wrist). The 2 stroke groups exhibited a similar lack of responsiveness to stimuli on their affected wrist after 1 week (see Fig 2B), and NT3 enhanced recovery compared to vehicle. Thus, NT3 improved responsiveness to tactile stimuli after stroke.

Walking was assessed using a horizontal ladder with irregularly spaced rungs (see Fig 2C). Accurate paw placement during crossing requires proprioceptive feedback from muscle spindles.^20^ After 1 week, the 2 stroke groups made a similar number of errors with their affected forelimb (see Fig 2D). NT3 caused a progressive recovery after stroke relative to vehicle‐treated animals. This is consistent with previous experiments from our laboratory.^14^, ^16^ Stroke also caused modest long‐lasting hindlimb deficits on the ladder; infusion of NT3 into the forelimb triceps brachii did not improve hindlimb movements (data in archive^27^).

NT3 also restored the use of the affected forelimb for lateral support when rats reared in a vertical cylinder (see Fig 2E). After stroke and vehicle treatment, rats used their affected forelimb less often than shams. NT3‐treated rats showed more frequent use of the affected forelimb relative to vehicle‐treated rats (see Fig 2F). Stroke caused transient weakness in forelimb grip strength in both groups, but infusion of NT3 into triceps brachii did not modify grip strength (data in archive^27^).

In summary, infusion of NT3 protein into the triceps brachii induced recovery on both sensory and motor tasks that require control of muscles by pathways including CSTs, serotonergic raphespinal pathways, and proprioceptive circuits. Accordingly, we hypothesized that NT3 would induce neuroplasticity in multiple pathways.

### *NT3 Induced Neuroplasticity in Multiple Spinal Locomotor Pathways*

We examined neuroplasticity in the C7 cervical spinal cord because we knew from experiments using adult and elderly rats that the less‐affected CST sprouts at this level after injection of AAV‐NT3 into muscles, including triceps brachii.^16^ Anterograde tracing from the less‐affected sensorimotor cortex (see Fig 1B) revealed that NT3 treatment increased sprouting of the CST in the C7 spinal cord (see Fig 3A, B) across the midline and into the affected side at 2 more lateral planes, and also from the ventral CST.

We assessed neural output in the ulnar nerve on the affected side, whose motor neurons are also found in C7 (range: C4–C8) and which supply muscles in the forearm, including the hand.^37^ To do this, we recorded responses during electrical stimulation of either the less‐affected CST or the partially ablated CST in the medullary pyramids. NT3 treatment led to enhanced responses in the ulnar nerve during stimulation of the less‐affected and more‐affected pathways (see Fig 3). This result is consistent with the sprouting of traced CST axons at C7 (see Fig 3A, B) and indicates that CST axons from both the stroke hemisphere and the contralesional hemisphere strengthened connectivity to the cord on the treated side, most likely on premotor interneurons that lie between CST axons and motoneurons. We also found that NT3 treatment caused serotonergic axons to sprout in the ventral C7 spinal cord (see Fig 4). Anatomical and functional plasticity of corticospinal and serotonergic pathways is consistent with their expression of receptors for NT3.^2^, ^3^

We conclude that NT3 caused neuroplasticity in multiple descending locomotor pathways. These data are consistent with our findings^14^, ^16^ that intramuscular AAV‐NT3 can, directly or indirectly, enhance spinal plasticity after stroke. Next, we assessed the biodistribution of NT3 after peripheral administration.

### *NT3 Enters the PNS and CNS after Peripheral Administration*

**Figure 5:**
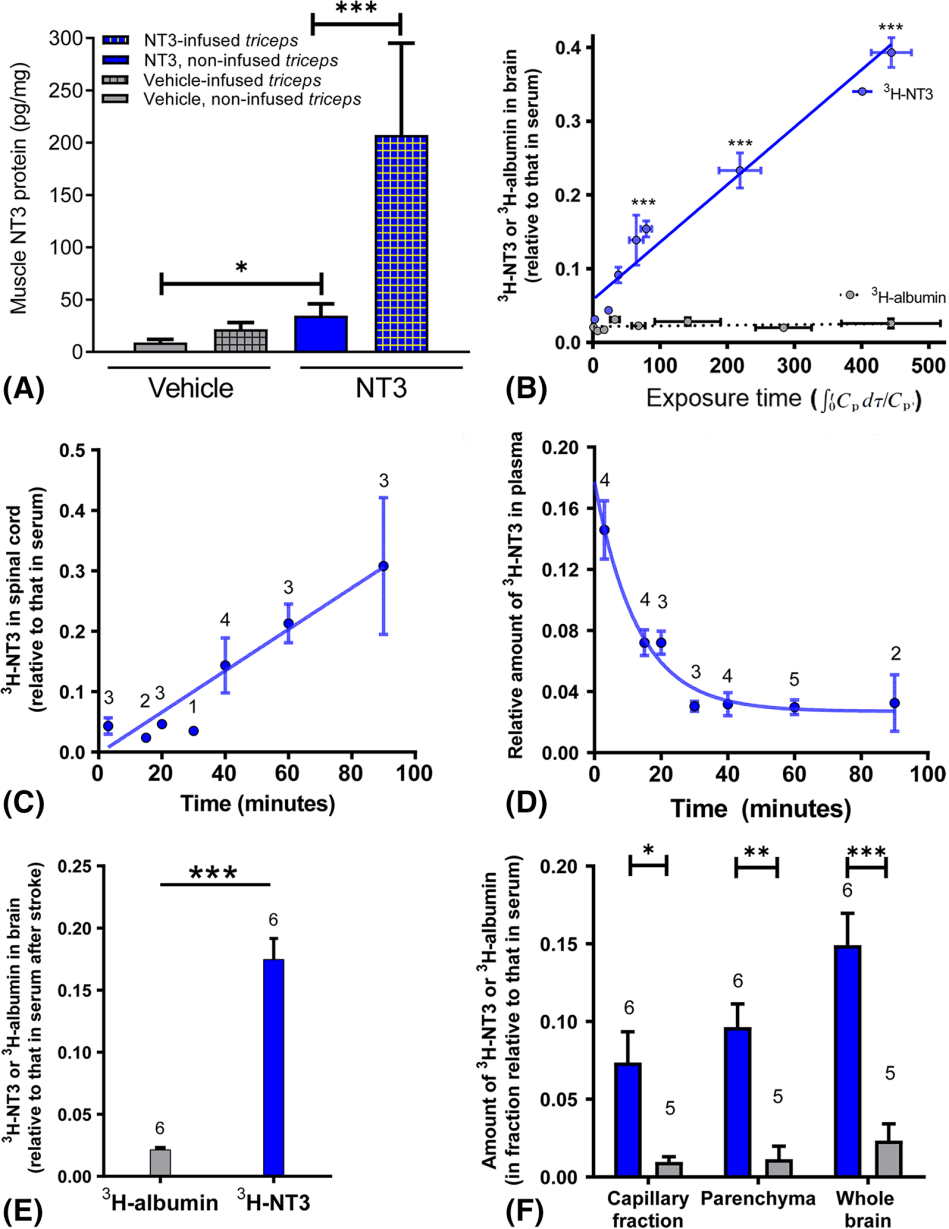
Levels of neurotrophin‐3 (NT3) were increased in triceps brachii, in brain, and in spinal cord. (A) Enzyme‐linked immunosorbent assay revealed that, in NT3‐treated rats, there was an increase in NT3 in the infused triceps brachii (Mann–Whitney vs vehicle, *p* = 0.032) as well as in the noninfused triceps brachii (Mann–Whitney vs vehicle, *p* = 0.032); n = 5 rats/group. (B) [^3^H]NT3 entered the brain more abundantly than [^3^H]albumin. *T* tests for NT3 versus albumin were all significant for times of 3, 15, 20 30, 40, 60, and 90 minutes (*p* values from <0.01 to <0.0001), which correspond to “exposure times” up to 450 minutes. Exposure time is calculated as ∫_0_^t^ *C*_*p*_(τ)dτ/*C*_*p*_, where *C*_*p*_ is concentration in plasma and τ is time in minutes. The graph is a Patlak plot^30^ showing regression of [^3^H]NT3 *(dark circles and continuous line)* and [^3^H]albumin *(light gray circles and dotted line)* against exposure time after intravenous (iv) injection (n = 2–5 mice/time) in adult mice. This corrects for the falling plasma concentration of solute with real time as shown in D. The volume of distribution (V_D_) of [^3^H]NT3 or [^3^H]albumin in brain is calculated as a ratio of counts per minute (CPM) in 1 μg of brain and CPM in 1 μl of serum for each time point and plotted against exposure time (rather than real time). The rate of influx (K_i_) was calculated from the Patlak plot^30^ of V_D_ for [^3^H]NT3 and [^3^H]albumin against the plasma area under the curve. At 1.3×10^−5^μl/mg/s, the unidirectional influx constant for NT3, K_i_, is 100 times that of the albumin (1.3 × 10^−7^ μl/mg/s). (C) [^3^H]NT3 entered the cervical spinal cord more abundantly than [^3^H]albumin. The graph shows real time and not exposure time. (D) Plasma half‐life of NT3 for the normal adult mouse is ~10 minutes (estimated from 1/normalized serum values). The graph shows real time and not exposure time. (E) Twenty‐four hours after cortical ischemia, [^3^H]NT3 entered the brain more abundantly than [^3^H]albumin, measured 40 minutes after iv injection. (F) Twenty‐four hours after cortical ischemia, levels of [^3^H]NT3 were higher in parenchyma depleted of endothelial cells than levels of [^3^H]albumin 40 minutes after iv injection (parenchyma *p* < 0.01, capillary fraction *p* < 0.05, and whole brain *p* < 0.001, 2‐way analysis of variance with Bonferroni post hoc tests). Results were corrected for the value of V_D_ for ^14^C sucrose. Number of mice per group is stated on panels. *p ≤ 0.05, **p ≤ 0.01, ***p ≤ 0.001. [Color figure can be viewed at www.annalsofneurology.org]

ELISA revealed an increase in total NT3 protein levels in the triceps brachii on the treated side (Fig 5A) and, surprisingly, on the untreated side (likely due to NT3 secreted into the bloodstream and taken up by endothelial cells; see below). We were not able to detect any small increases in exogenous human NT3 against the background of endogenous rat NT3 in the C7 spinal cord (data in archive^27^), because the amino acid sequences for mature human and rat NT3 are identical.^38^ Accordingly, we next used a more sensitive method for measuring trafficking of exogenous NT3 across the blood–CNS barrier.

Radiolabeled NT3 was injected intravenously into adult mice. Radiolabeled albumin was used as a control, because it does not enter the CNS efficiently from the bloodstream. After 3, 15, 20, 30, 40, 60, or 90 minutes, brain, spinal cord, and serum were taken for scintillation counting. [^3^H]NT3 progressively accumulated in the intact brain and cervical spinal cord (see Fig 5). In plasma, the half‐life of NT3 was short. Our data are consistent with those of others^22^, ^23^, ^24^; for example, after injection of radiolabeled NT3 into the brachial vein (which provides drainage from the triceps brachii), NT3 accumulates in the cortex, striatum, brainstem, cerebellum, spinal cord, and sciatic nerve, although the majority of NT3 is cleared rapidly from the bloodstream.^22^, ^23^ Moreover, the blood–nerve barrier in DRGs is fenestrated and permeable to proteins like NT3.^39^

Next, [^3^H]NT3 or [^3^H]albumin was injected intravenously into adult mice 24 hours after cortical ischemia. Forty minutes later, tissues were taken for scintillation counting. Again, [^3^H]NT3 accumulated in the brain (see Fig 5E). To confirm entry of [^3^H]NT3 into brain parenchyma beyond endothelial cells, capillaries were depleted by gradient centrifugation to yield a fraction containing brain parenchyma and an endothelial cell–enriched pellet. [^3^H]NT3 entered parenchyma (depleted of endothelial cells) at a level above that seen for [^3^H]albumin (see Fig 5F). Transport of NT3 into the CNS is apparently a receptor‐mediated process.^6^, ^40^

We conclude that neuroplasticity occurred in multiple locomotor pathways because peripherally administered NT3 bound to receptors in the PNS and CNS.

### *Blood Oxygenation Level–Dependent Signal in Perilesional Somatosensory Cortex Evoked by Stimulation of the Affected Wrist Recovers with Time after Stroke and Is Not Further Enhanced by NT3*

**Figure 6:**
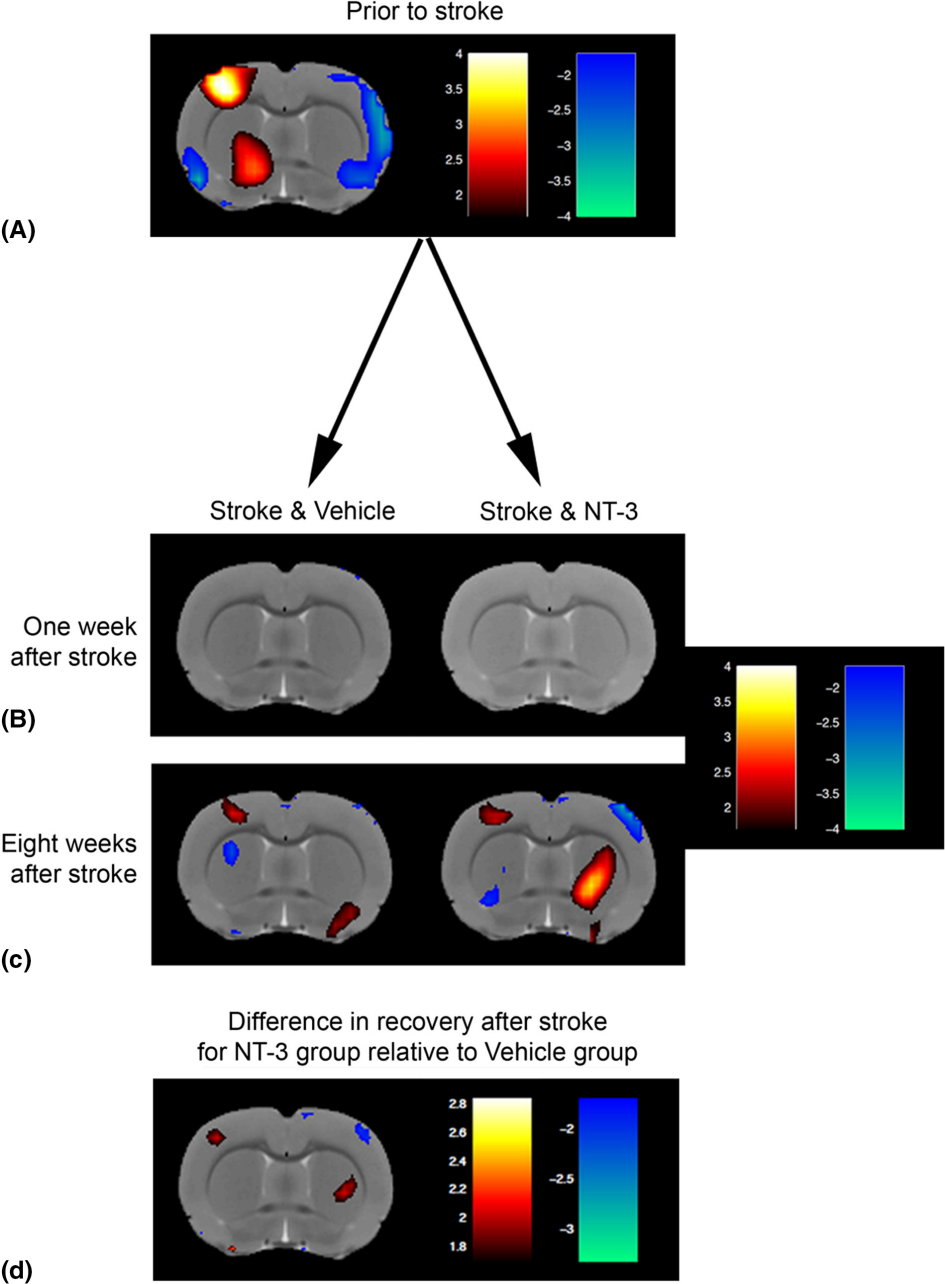
Functional magnetic resonance imaging during stimulation of the affected wrist revealed increased probability of perilesional reactivation in both stroke groups but no additional increase by neurotrophin‐3 (NT3). The same rats were imaged prior to stroke and then 1 week and 8 weeks after stroke and intramuscular treatment with either NT3 or vehicle. Scans with obvious imaging artifacts were discarded, leaving final group numbers of n = 7, 9, and 7 and n = 9, 8, and 4 at weeks 0, 1, and 8 for the NT3‐treated and vehicle‐treated groups, respectively. Red voxels denote greater probability of activation during stimulation (vs stimulation off), whereas blue voxels denote lesser probability of activation during stimulation (vs stimulation off). (A) Prior to stroke, stimulation of the dominant paw led to a strong probability of activation in the opposite somatosensory cortex. (B) One week after stroke, this activation was abolished by infarction. (C) Eight weeks after stroke, there was a slight trend toward a small perilesional area of reactivation in both groups. (D) There was a slight trend toward greater perilesional reactivation in the NT3 group versus the vehicle group at 8 weeks (relative to their baselines). However, all these heat maps of group mean activations show *t* values obtained by Statistical Parametric Mapping analysis without correction for multiple testing (*p* < 0.05) and there were no differences between the 2 groups for any voxels when the threshold for significance was corrected for multiple testing (*p* < 0.01; these data are not shown, as the heat map was black). Red voxels denote greater probability of activation during stimulation for the NT3 group than for the vehicle group, whereas blue voxels denote lesser probability of activation for the NT3 group than for the vehicle group. During stimulation of the less‐affected wrist, there were no differences between the 2 groups for any voxels when the threshold for significance was corrected for multiple testing (*p* < 0.01; these data are not shown, as the heat map was black).

To explore the mechanism whereby NT3 improved responsiveness to stimuli attached to the affected wrist (see Fig 2B), we performed blood oxygenation level–dependent (BOLD)‐fMRI during low intensity (non‐noxious) electrical stimulation of the affected wrist (Fig 6). As expected, prior to stroke, stimulation of the wrist resulted in a higher probability of activation of the opposite somatosensory cortex. One week after stroke, somatosensory cortex was not active when the affected paw was stimulated in either vehicle‐treated or NT3‐treated rats (*p* values >0.05). fMRI performed 8 weeks after stroke revealed a trend toward perilesional reactivation of somatosensory cortex in both vehicle‐treated and NT3‐treated groups (*p* values <0.05). However, these probabilities of reactivation were not big enough to survive correction for testing of multiple voxels (threshold = 0.01).

A longitudinal analysis showed that at 8 weeks, there was some evidence that rats treated with NT3 showed increased probability of activation of perilesional cortex (*p* values <0.05; see Fig 6D) and showed decreased probability of activation of cortex in the less‐affected hemisphere (*p* values <0.05) relative to vehicle‐treated stroke rats. However, these apparent differences did not survive correction for testing of multiple voxels (threshold = 0.01). We conclude that both groups showed partial, spontaneous restoration of more‐normal patterns of somatosensory cortex activation^41^ but, conservatively, that NT3 did not further increase probability of activation of any supraspinal areas. These conclusions are consistent with previous fMRI data from our laboratory;^16^ we propose that the additional recovery of somatosensory function after NT3 treatment (see Fig 2B) is due to changes in the spinal cord rather than in supraspinal areas.

### *NT3 Did Not Cause Detectable Hypersensitivity*

**Figure 7:**
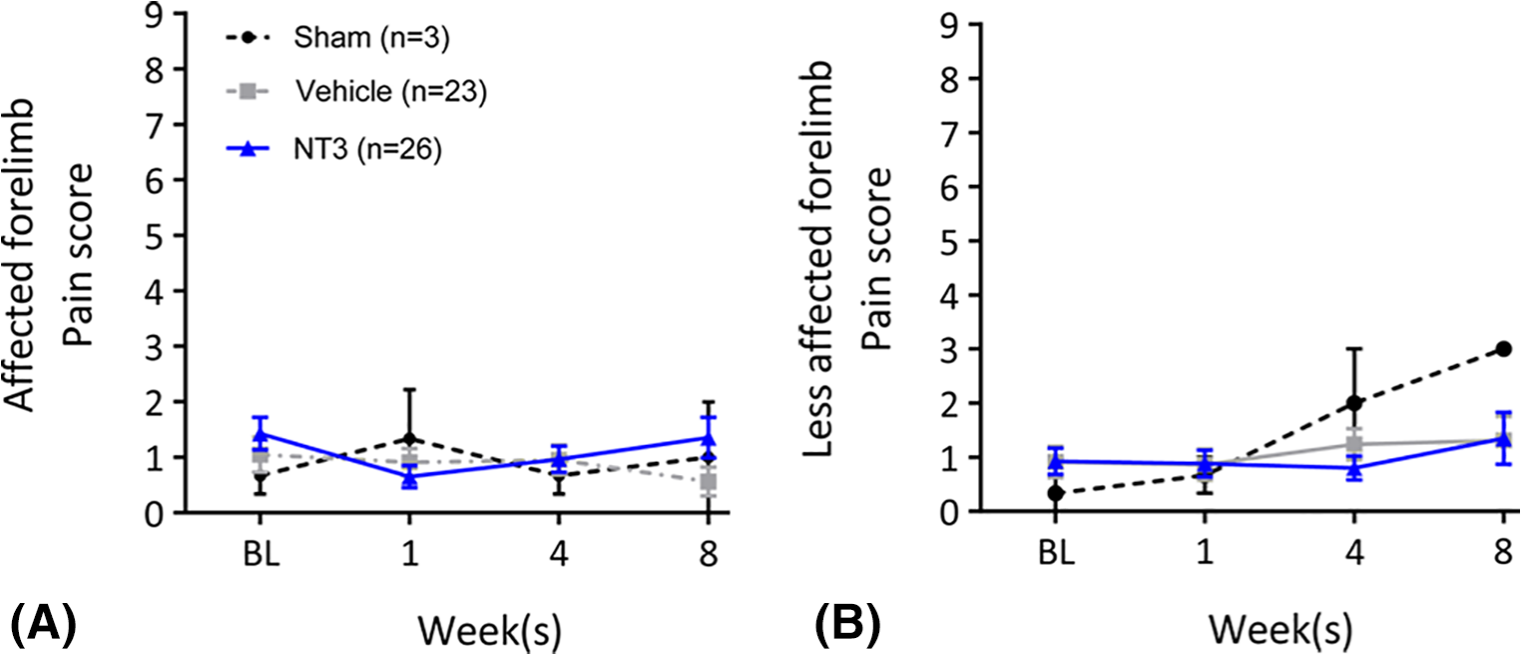
Cold allodynia was caused neither by focal cortical stroke nor by treatment with neurotrophin‐3 (NT3). The acetone test was used to determine whether stroke and/or NT3 treatment caused any change in cold allodynia pain responses. The test involves applying a drop of acetone to the (A) affected or (B) less‐affected forelimb, and then allocating a score between 0 and 9; higher numbers denote a heightened pain response. There is no evidence of painful behavior based on this test in either forelimb. Repeated measures analysis of covariance was used with Bonferroni post hoc tests. [Color figure can be viewed at www.annalsofneurology.org]

Finally, we also found no evidence that stroke or NT3 caused hypersensitivity in the forepaw, assessed by application of ice‐cold acetone (Fig 7). This is consistent with our previous work showing a lack of mechanical hyperalgesia after NT3 treatment,^14^ with the lack of TrkC on adult nociceptors,^5^ and with the human clinical trial data showing that subcutaneous treatment with NT3 was safe and well tolerated.^25^, ^26^

## Discussion

Intramuscular infusion of NT3 protein, initiated 24 hours after stroke, caused changes in multiple locomotor circuits, and promoted a progressive recovery of sensorimotor function in rats. That NT3 can reverse disability when treatment is initiated 24 hours after stroke is exciting, because most stroke victims are diagnosed within this time frame. NT3 could potentially be used to treat many survivors.

NT3 has good clinical potential. First, phase II clinical trials show that doses up to 150 μg/kg/day are well tolerated and safe in healthy humans and in humans with other conditions.^25^, ^26^ We used a 3‐fold lower dose (48 μg/kg/day) in this study; in future experiments, we will optimize the dose and duration of treatment, because it is possible that a higher dose of NT3 would promote additional recovery. Second, there is conservation from rodents to humans in the expression of receptors for NT3 in the locomotor system.^2^, ^3^ Third, in none of our rodent experiments has NT3 treatment caused any detectable hypersensitivity, spasticity, or muscle weakness; rather, after bilateral CST injury in rats, intramuscular delivery of AAV‐preproNT3 reduced spasms, slightly improved grip strength, and showed a trend toward reducing mechanical hyperalgesia.^14^

We used fMRI during electrical stimulation of the wrist to explore recovery of somatosensory responsiveness to adhesive patches attached to the wrist. We confirmed work by others that spontaneous recovery correlated well with more normal patterns of increased BOLD signal surrounding the infarct^41^, ^42^ but, as before,^16^ we did not find strong evidence that NT3 further increased perilesional (or other) activation. Accordingly, we propose a different hypothesis: that NT3 increased somatosensory recovery^27^ by inducing neuroplasticity in spinal circuits involving cutaneous afferents. Using a different animal model, we have previously shown that NT3 normalizes output from spinal circuits following stimulation of low‐threshold afferents from the treated wrist^14^ (which would activate cutaneous afferents as well as proprioceptive afferents, both of which express receptors for NT3^5^). However, in those experiments, we measured output in motor axons rather than sensory output, so in the future one might examine whether NT3 modulates gating of somatosensory inputs from the wrist^43^ after stroke.

With regard to corticospinal neuroplasticity, we have shown twice previously (in adult and elderly rats) that the less‐affected CST sprouts across the cervical midline into the affected hemicord after injection of AAV‐NT3 into affected forelimb muscles.^16^ Others have shown that intrathecal infusion of NT3 induces sprouting of the CSTs^13^ and that injection of vectors encoding NT3 into muscles^8^ or nerve^9^ induces CST sprouting after spinal cord injury. Here, our anatomical tracing confirmed that the less‐affected CST sprouted after infusion of NT3 protein into triceps, and in future we will trace both CSTs. This is because, in the present study, neurophysiology revealed that both CSTs underwent plasticity to the affected side after unilateral infusion of NT3 protein.

We propose that spared CST axons sprouted after NT3 entered the CNS from the systemic circulation. This is consistent with data from us and others showing that radiolabeled NT3 entered the brain and spinal cord after intravenous injection.^22^, ^23^, ^24^ Moreover, it has been shown that endogenous muscle spindle–derived cues induce sprouting of descending pathways after spinal cord injury in adult mice^20^; given that muscle spindles make NT3 endogenously,^21^ it is plausible that infusion of supplementary NT3 to muscle might directly enhance CST sprouting after stroke via TrkC receptors on corticospinal axons.^3^, ^44^

It is possible, additionally, that NT3 was trafficked from forelimb muscles in axons to motor neurons and/or to DRG neurons, where it induced expression of a molecule that was secreted and induced CST sprouting. NT3 is certainly trafficked to ipsilateral motor neurons and DRGs after intramuscular delivery.^4^, ^6^, ^14^, ^16^, ^40^ Diffusion within neuropil is inefficient,^45^ but spinal motor neuron dendritic arbors can be very large (some even extend across the midline^46^), and these might widely distribute a cue for supraspinal axonal plasticity (eg, across the midline). In another project, to seek DRG‐derived factors, we have performed RNAseq of cervical DRGs after injection of AAV‐NT3 into forelimb flexors.^14^, ^47^ In the future, we will also seek motor neuron–derived cues that are regulated by NT3.

Unilateral stroke often causes bilateral impairments in humans and other mammals.^48^ In principle, intramuscular infusion of NT3 into an affected muscle might (after distribution of NT3 in the bloodstream) enhance sprouting of CST axons to the less‐affected side (as well as the affected side), thereby increasing output to forelimb motor nerves in the less‐affected limb. However, impairments on the less‐affected side are modest and transient in rats with this model of stroke,^27^, ^32^ and so any neuroplasticity might be difficult to detect. It is also notable that infusion of NT3 into a proximal forelimb extensor improved the accuracy of use of the affected forelimb during walking on a horizontal ladder but did not improve the accuracy of the affected hindlimb (see Duricki et al^27^); future studies will determine whether higher doses of circulating NT3 might improve hindlimb movements. Moreover, infusion of NT3 protein into triceps brachii did not improve forelimb grip strength;^27^ however, the grip strength task probably depends more on strength in hand and digit flexor muscles (into which NT3 was not infused) than on triceps brachii (elbow extensors). Injection of AAV‐NT3 into proximal and distal flexor forelimb muscles does modestly improve grip strength.^14^ Taken together, these results indicate that it may be strategic to target NT3 to multiple muscles.

Finally, it is encouraging, from a translational perspective, that the recovery continues even after infusion of NT3 is discontinued at 4 weeks. We propose that the 4‐week–long NT3 treatment induces changes in target cells that persist (eg, due to sustained modifications in gene expression). Longer treatment with NT3 induces different intracellular signaling events in sensory axons than does brief treatment, thereby enhancing terminal branching.^49^ We are currently seeking factors that are persistently increased in target neurons after NT3 treatment is discontinued. Additionally, it may be that NT3 induces sprouting of CST axons that (after cessation of treatment) is followed by selection of synapses (eg, strengthening or pruning) by a mechanism that is independent of NT3. For example, it is known that corticomotoneuronal axon synapses are pruned by repulsive PlexinA1–Sema6D interactions.^50^ To dissect the mechanisms whereby NT3 promotes neuroplasticity and recovery after peripheral delivery, we have set up a mouse model of stroke.

In summary, treatment of impaired arm muscles with NT3 (initiated in a clinically feasible time frame) induces spinal neuroplasticity, improves walking, and reverses a tactile sensory impairment. Other experimental therapies that can be delivered with a delay after stroke include antibodies against Nogo‐A, which also induce neuroplasticity of cortical efferents.^51^, ^52^, ^53^ Given the importance of the CST for spontaneous recovery after stroke,^54^ these novel strategies offer encouraging prospects for enhancing neural repair.

## Author Contributions

D.A.D. and L.D.F.M. contributed to the conception and design of the study; all authors contributed to acquisition and analysis of data; D.A.D., L.D.F.M., S.D., and T.W. contributed to drafting the text and preparing the figures.

## Potential Conflicts of Interest

Nothing to report.
